# Low-technology cooling box for storage of malaria RDTs and other medical supplies in remote areas

**DOI:** 10.1186/1475-2875-9-31

**Published:** 2010-01-23

**Authors:** Lon Chanthap, Frédéric Ariey, Duong Socheat, Reiko Tsuyuoka, David Bell

**Affiliations:** 1 National Center for Parasitology, Entomology and Malaria Control, #372 Blv Monivong, Phnom Penh, Cambodia; 2 Laboratory of Molecular Epidemiology Pasteur Institute of Cambodia, #5, Monivong Blvd, PO Box 983, Phnom Penh, Cambodia; 3 Communicable Diseases Surveillance and Response, World Health Organization, Ban Phonexay, That Luang Road, PO Box 343, Vientiane, Lao PDR; 4 Foundation for Innovative New Diagnostics (FIND), 16 Avenue de Budé, 1202 Geneva, Switzerland (Formerly World Health Organization - Regional Office for the Western Pacific)

## Abstract

**Background:**

With the increase in use of point-of-care diagnostic tests for malaria and other diseases comes the necessity of storing the diagnostic kits and the drugs required for subsequent management, in remote areas, where temperatures are high and electricity supply is unreliable or unavailable.

**Methods:**

To address the lack of temperature-controlled storage during the introduction of community-based malaria management in Cambodia, the Cambodian National Centre for Parasitology, Entomology and Malaria Control (CNM) developed prototype evaporative cooling boxes (Cambodian Cooler Boxes - CCBs) for storage of perishable medical commodities in remote clinics. The performance of these CCBs for maintaining suitable storage temperatures was evaluated over two phases in 2005 and 2006-7, comparing conditions in CCBs using water as designed, CCBs with no water for evaporation, and ambient storage room temperatures. Temperature and humidity was monitored, together with the capacity of the RDTs recommended for storage between 2 to 30 degree Celsius to detect low-density malaria parasite samples after storage under these conditions.

**Results:**

Significant differences were recorded between the proportion of temperatures within the recommended RDT storage conditions in the CCBs with water and the temperatures in the storage room (p < 0.001) and maximum temperatures were lower. RDTs stored at ambient temperatures were negative when tested with parasitized blood (2,000 parasites per micro litre) at 210 days, while the field RDTs kept in CCBs with water gave positive results until 360 days.

**Discussion and Conclusions:**

The CCB was an effective tool for storage of RDTs at optimal conditions, and extended the effective life-span of the tests. The concept of evaporative cooling has potential to greatly enhance access to perishable diagnostics and medicines in remote communities, as it allows prolonged storage at low cost using locally-available materials, in the absence of electricity.

## Background

Since malaria rapid diagnostic tests (RDTs) were introduced in Cambodia in 1996, the use of the tests has increased steadily. The Cambodian programme uses RDTs to improve diagnosis of febrile illness in remote malaria-endemic areas where microscopy diagnostic services are not readily available. Rising treatment costs due to introduction of artemisinin-combination therapy (ACT) has further raised the importance of proper malaria diagnosis prior to treatment, and the urgency to reduce and eliminate emerging artemisinin-resistant malaria in western Cambodia has made rapid access, at community level, to both RDTs and anti-malarial drugs a priority for the Cambodian National Centre for Parasitology, Entomology and Malaria Control (CNM).

Malaria RDTs are lateral-flow tests based on interactions of biological agents (antibodies and antigens) attached to or flowing along a nitro-cellulose strip. They are therefore sensitive to degradation by heat and humidity. Heat can damage RDTs through deconjugation of the antibody-dye conjugate, detachment of the bound antibody from the nitrocellulose, loss of ability of the antibody to bind to antigen, and degradation of the nitrocellulose strip. While exposure to humidity can be prevented by packaging in moisture-proof envelopes, if RDTs are stored at temperatures exceeding the recommended temperature it is likely that loss of sensitivity will occur and the shelf-life of the RDTs will be reduced [1].

Previous studies in Cambodia have demonstrated storage temperatures for RDTs much higher than 30°C in remote health facilities. Temperatures in drug storerooms in some health centres can reach 42.5°C [2].

CNM, with support from the WHO-Regional Office for the Western Pacific, has pioneered the introduction of evaporative cooler boxes for storage of medical supplies in remote clinics in Cambodia, known locally as the "Cambodian Cooler Box" (CCB). Cooling is based on the principle that water absorbs heat from its surroundings when changing from a liquid phase to its higher energy gaseous phase (evaporation). As heat is absorbed from the surroundings of the damp sacking cover, particularly from the galvanized iron sides of the box with which the sacking is in direct contact, the box sides and contents are cooled [3]. The same principle has been used since antiquity to cool water and preserve food in hot climates, and is still used for this purpose, using sacking or porous terracotta vessels, in parts of the tropics and sub-tropics today [4-7].

The study reported here aimed to: (1) investigate the difference in temperature and humidity inside and outside the cooler boxes in the health facility; (2) investigate the effect of water on CCB function independent of other aspects of its design; and (3) investigate the difference in performance of RDTs stored inside the cooler boxes and those kept in the storage room of the health facility at ambient temperatures.

## Methods

This prospective study was done in two phases. Phase 1 was a pilot study conducted in Sampov Loun operational district storeroom (SL) and Ankor Ban Health Centre (AB) in Battambang province, Cambodia, from May to July, 2004. The province has a clearly defined dry season (December to May) and rainy season (June to November). The maximum temperature in the dry season can reach 38°C. This phase of the study was designed to demonstrate the temperature and humidity differences in the storerooms at ambient temperature, in the CCB without water and in the CCB with water.

Phase 2 was conducted in SL, Serey Meanchey health centre storeroom (SM) and, Barieng Tlek storeroom (BT) in Battambang province, Cambodia between November 2005 and December 2006. These areas are malaria-endemic for both *Plasmodium falciparum *and *Plasmodium vivax*, and RDTs use used for malaria diagnosis. This phase of the study focused on investigation of temperature and humidity to which the RDTs were exposed and the stability of the RDTs during exposure to temperature and humidity in three conditions; ambient room temperature, CCB without water and, CCB with water (as designed).

**Figure 1:**
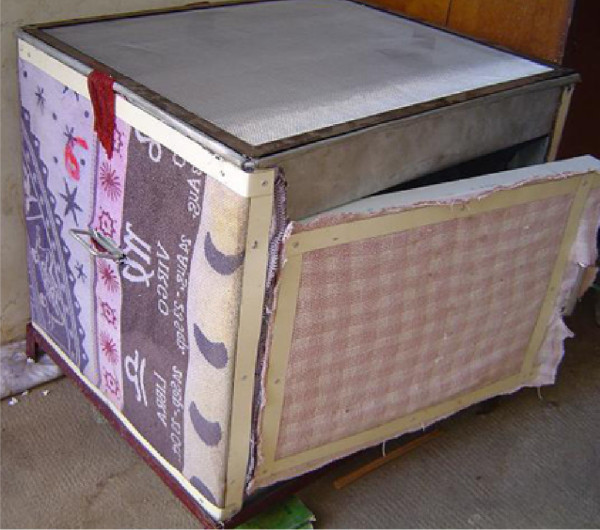
**Photo of 'Cambodian Cooling Box' (CCB)**. A cloth wick connects the water reservoir contents to the cloth sides except on the side with the door

**Figure 2:**
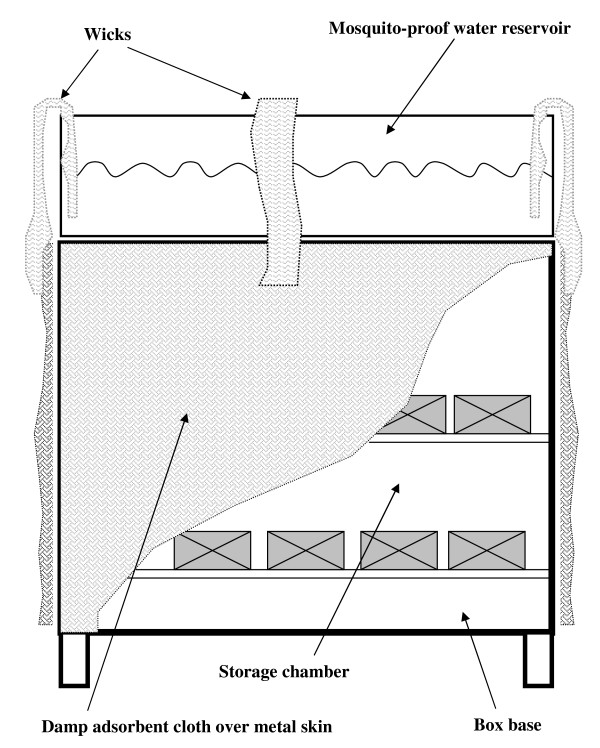
**Diagram of the cooler**.

The prototype CCB used in this study is an evaporative cooler box, made of 1 mm thick galvanized iron sheet and has a volume of 24 litres (L) (Figure 1). A tray on top stores 9 L of water as water source. The tray is covered by mosquito-proof mesh. Local cotton sack material (0.5 cm thick) covers all outer surfaces including a side door. All sides are connected to the water source through cheesecloth bands (wicks) that draw water from the reservoir onto the sacking on the box sides by capillary action. The flow will thus increase when the sacking is drier and capillary effect greater (Figure 2).

To compare effectiveness and temperature inside CCB and ambient temperature in storeroom, two CCBs (with and without water) were placed together in the same storerooms. Local health centre staff daily monitored and recorded relative humidity three-times a day, verified soaking of cloth surrounding of CCB, and added water to the tray. Bi-monthly supervision using check lists was performed by the study team and temperature devices were replaced every 3 months, before the internal memories were full.

Temperature and humidity were monitored using a temperature monitoring device (TM) (Smart Button Temperature Logger; ACR Systems, Surrey, Canada; range -10°C to +85°C, accuracy ± 0.5°C), and a thermohygrometer (Hygrometer testo 608-H1; Probe type "NTC humidity sensor"; Germany; range 10 to 95% RH, accuracy ± 3% RH) stored in each CCB and in the storerooms. The temperature was automatically recorded by the TM every one hour and relative humidity measured by the hygrometer was manually recorded by local staff in the morning (8 h 00-9 h 00), midday (11 h 00-13 h 00) and early evening (16 h 00-18 h 00). A supervisor checked each site every two months. One hundred and fifty *Plasmodium *lactate dehydrogenase (pLDH) - detecting RDTs within their expiry date of November 2006 (Diamed IT, Diamed, Switzerland, lot number 46110.78.01) were stored under each condition at each site. Nine RDTs were randomly retrieved from each condition monthly in every location.

Testing of RDTs was performed by the malaria RDT quality control laboratory of the Institut Pasteur du Cambodge (IPC) following the WHO protocol (Methods Manual for Laboratory Quality Control Testing of Malaria Rapid Diagnosis Tests)[8]. The RDTs were tested with negative and positive blood from the sample bank of the IPC malaria RDT quality control laboratory [sample ID: C2N (negative bank) and C2F15 (200, 500 and, 2000 parasites per micro liter-μL)]. A product-specific rating card was used to grade line intensity as 0 (negative), 1+, 2+, 3+ or 4+ for each product tested, to ensure consistency of each reading testing. Testing was ceased if negative test results were obtained at two consecutive sampling intervals.

### Training, supervision and monitoring

Before the start of project implementation, all study team members were trained on recording the information on Data Collection Forms. Bi-monthly supervision of field staff by the Principal Investigators was done to assess quality of recorded data using checklists, to replace TM devices, retrieve Pf-LDH RDT samples and to collect other materials. All samples and a TM were kept in a vaccination cooler box with dry ice during the transport samples from the field. Local staff members were trained to check the CCBs regularly. Then the volume of added water was recorded and the water storage tray was filled up to the maximum mark line, 3/4 height of the tray, 2-3 times a week.

### Data analysis

Temperature data was analysed using SPSS version 12.0.1 (SPSS Inc., 2004, Chicago, IL, USA), comparing the three storage conditions. The relative humidity data were analysed using Microsoft Excel to compare average humidity for the three conditions. The proportion of temperatures above 30°C was compared using Chi-square tests.

## Results

**Figure 3:**
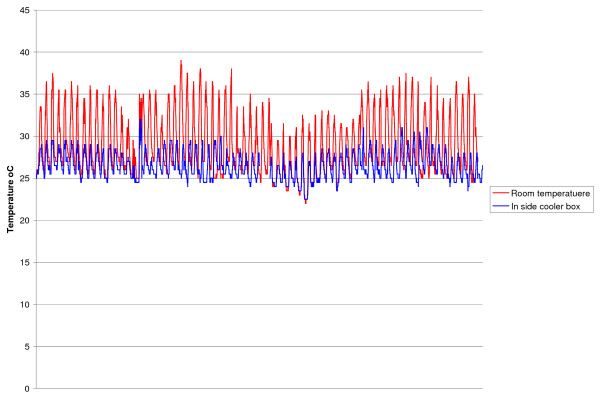
**Comparison of temperature in room and cooler box with water Angkor Ban Health Centre 2004**.

In Phase 1, the mean ambient storeroom temperatures were 28.9°C (range 22-39°C) and 27.7°C (range 23-33.5°C) in AB and SL, respectively. Temperatures more than 30°C in the storerooms were for 32% and 9.3% of the total number of monitored hours (hrs) in AB and SL respectively, while temperatures more than 30°C inside CCBs with water were 1.1% and 0.3% in AB and SL, respectively. There were significant differences between the proportion of temperatures more than 30°C in the CCB with water compared with the corresponding temperature in the storage room (p < 0.001) in both sites while the cooler box without water provided some protection from high temperatures (Table 1 and Figure 3).

**Table 1:** Cumulative hours of temperature > 30°C in Cambodian Cooler Boxes and in the storeroom: phase 1

Location	Experimental condition	Total hours of monitoring	Hours > 30°C	p*
				
			n (% of total)	
**Sampour Lo**	1. In Cambodian Cooler Box with water	6,788	18 (0.3)	0.001
	
	2. In Cambodian Cooler Box without water	6,788	305 (4.5)	0.010
	
	3. Storage Room	6,788	631 (9.3)	

**Ankor Ban**	1. In Cambodian Cooler Box with water	6,587	75 (1.1)	0.001
	
	2. In Cambodian Cooler Box without water	6,587	360 (5.4)	0.04
	
	3. Storage Room	6,588	2,106 (32)	

* Chi-square test

In Phase 2, the mean ambient storeroom temperatures were 26.6°C (range 16 - 33°C), 26.7°C (range 17.5 - 33°C) and, 26.6°C (range 14.5 - 33.5°C) at SL, BT and, SC storerooms respectively. The mean temperature in the CCBs without water were 26.4°C (range 17 - 32.5°C), 26.2°C (range 16 - 32°C) and, 26.1°C (15 - 32.5°C) at SL, BT and, SC, respectively and the mean temperature in the CCBs with water were 25.1°C (range 16.5 - 31°C), 25.2°C (range 15 - 32°C) and, 26.1°C (15 - 31.5°C) at SL, BT and, SC, respectively. Temperatures more than 30°C in the storerooms were recorded for 3.6%, 5.1% and 4.9% of the total number of monitoring hrs in SL, SC and, BT, respectively. During of study, temperatures higher than 30°C inside CCBs with water occurred for 0.1% to 0.2% of the time in SL, SC and, BT (Table 2). The highest frequency of temperatures above 30°C in the ambient storerooms was observed from January to June 2006 in all sites. There are significant differences between the proportion of temperatures higher than 30°C in CCB with water compared with temperatures in the storage room (p < 0.001) in the three sites. However, there was no significant difference between the proportion of temperatures higher than 30°C in the CCB without water compared temperatures in the storage room (p = 0.154 and 155) in SC and BT (Table 2).

**Table 2:** Cumulative hours of temperature > 30°C in Cambodian Cooler Boxes and in the storeroom: Phase 2

Location	Experimental condition	Total hours of monitoring	Hours > 30°C	P*
				
			n (% of total)	
**Sampour Lon**	1. In Cambodian Cooler Box with water	10,610	6 (0.1)	0.001
	
	2. In Cambodian Cooler Box without water	10,610	41 (0.4)	0.004
	
	3. Storage Room	10,610	378 (3.6)	

**Barieng Tlek**	1. In Cambodian Cooler Box with water	10,608	18 (0.2)	0.001
	
	2. In Cambodian Cooler Box without water	10,608	189 (1.8)	0.009
	
	3. Storage Room	9,546	485 (5.1)	

**SC**	1. In Cambodian Cooler Box with water	10,606	9 (0.1)	0.001
	
	2. In Cambodian Cooler Box without water	10,606	189 (1.8)	0.009
	
	3. Storage Room^3^	9,264	451 (4.9)	

* Chi-square test

The average related humidity (RH) in CCBs with water ranged from 75% to 85% in mornings and in evenings, 5-15% higher than RH in the ambient storeroom and CCB without water and with less fluctuation (Table 3).

**Table 3:** Average relative humidity in % in different storage conditions in 3 health facility locations

Location	Experimental condition	Average relative humidity in % at Morning (8:00 to 9:00)	Average relative humidity in % at Afternoon (11:00-13:00)	Average relative humidity in % at Evening (4:00-6:00)
**Sampour Lon**	1. In Cambodian Cooler Box with water	85.0	84.4	83.6
	
	2. In Cambodian Cooler Box without water	72.6	71.9	71.4
	
	3. Storage Room	78.2	70.1	68.2

**BRT HC**	1. In Cambodian Cooler Box with water	80.8	80.3	76.5
	2. In Cambodian Cooler Box without water	73.3	72.3	71.6
	3. Storage Room	77.9	72.2	70.6

**Serem Meanchey Health Centre**	1. In Cambodian Cooler Box with water	80.7	80.8	80.2
	
	2. In Cambodian Cooler Box without water	74.1	73.8	74.3
	
	3. Storage Room	80.3	78.0	71.4

The batch of RDT used in the study did not consistently detect lower dilutions prepared by IPC at baseline, so only detection of 2,000 parasite/μL is discussed here. The density of the *P. falciparum *positive test line of RDTs kept at ambient storeroom temperatures and in the CCBs without water decreased from 2+ to 1+ when tested on the samples at 2,000 parasite/μL, while the field RDTs kept in the CCBs with water remained at 2+ intensity at 120 days of field monitoring period. The RDTs stored under 28°C at IPC remained 2+ at 120 days. At 210 days, field RDTs kept in ambient temperature in storerooms and in the CCBs without water were negative (no antigen detected), while the field RDTs kept in the CCBs with water remained positive with an intensity of 1+. The field RDTs kept in the CCBs with water eventually became negative at 300 days at the SM and BT sites and, at 360 days at the SL site.

## Discussion

Maintenance of perishable medical supplies within their specified storage temperatures is essential to maintaining the quality of, and confidence in, malaria RDTs [1] as it is with other diagnostic devices and with medicines. Previous studies have shown that simulated high temperatures have a significant impact on performance during the shelf-life of some RDTs [9] and reduced sensitivity or failure of RDTs may occur due to exposure to excessive temperatures [1,10]. This has often been seen as an impediment to improving access of remote and rural populations to health interventions, particularly for pLDH and aldolase-detecting RDTs. This study has demonstrated that this impediment can be overcome for malaria case management through the use of simple, low-cost and locally-available technology. Despite clinic storage temperatures rising frequently above the manufacturers specified storage temperatures, the evaporative cooler box maintained the malaria RDTs within the required range for all but a few occasions, greatly extending the time over which the RDTs detected the parasite samples.

Evaporative cooling is an ancient technology that is still used for cooling water, and various coolers working on similar designs to the ones used in this study have been designed for maintaining cool temperatures for food storage [11,12]. While the idea is not new, the principle has not been put to use on a large scale to allow extension of important health interventions. The CCB used local materials and construction, and the prototypes cost only USD 25.00 to produce at that time. Significant cooling was achieved despite Cambodia being cooler than many other malaria-endemic countries and having high humidity, both of which should reduce the relative efficiency of evaporative cooling. The second phase of this study also took place across an unusually mild 'hot season'.

The relative humidity in CCB having water in the study showed an increase of up to 10% from the humidity in the general clinic storage areas. However; there is unlikely to be any significant impact on performance of the products, as most RDT manufacturers use hermetically sealed packets and a desiccant to protect the test devices.

Development of a "cool chain" is not only important for many RDTs [13,14], it is also important for many medicines. Medicines are commonly stored and transported under the same conditions as RDTs in developing countries. In general, medicines should be stored under conditions similar to RDTs. Coartem^® ^(artemether-lumefantrine) has a recommended storage temperature not exceeding 30°C.

## Conclusions

The study demonstrated that a low-cost, locally-produced product employing evaporative cooling technology can effectively maintain safe storage temperatures well-below room temperatures in tropical conditions, even in the presence of relatively high ambient humidity. This has applications for the maintenance of quality of diagnostics and drugs in clinics in village-based settings. Further studies to further improve of design of the CCB are warranted since optimization of sacking materials, volume to surface area ratios and floor insulation should further improve efficiency. As a consequence of this study, CNM and partners in Cambodia are currently introducing, over the coming year, more than 1,200 evaporative cooling boxes into western Cambodia, in remote health facilities where basic utilities, including electricity, are lacking.

## Conflict of interests

The authors declare that they have no competing interests.

## Authors' contributions

CL: co-designed the study, monitored in the field, analysed data and drafted the report and prepared the manuscripts. SD: co-designed and approval the study procedure and follow data collection. FA: Laboratory analysis test. RT: co-designed the study, monitored in the field, analysed data and co-ordination. DRB: approved the study design, analysed data and finalized the manuscripts and funding.

The project was supported by WHO-Regional Office for the Western Pacific.

All authors have read and approved the final manuscript.
